# P-403. Incidence of Surgical Site Infections in Selected Tertiary Care Hospitals in Bangladesh: Findings From A Pilot Surveillance Project

**DOI:** 10.1093/ofid/ofae631.604

**Published:** 2025-01-29

**Authors:** Md Golam Dostogir Harun, Md Shariful Amin Sumon, Md Saiful Islam

**Affiliations:** icddrb, Dhaka, Dhaka, Bangladesh; icddr,b, Dhaka, Dhaka, Bangladesh; University of New South Wales, Sydney, South Australia, Australia

## Abstract

**Background:**

Surgical site infections (SSI) are the most frequent type of healthcare-associated infections (HAI) and SSIs have substantial healthcare ramifications. In Bangladesh, most hospitals lack surveillance systems for HAIs, including SSI. We conducted this SSI surveillance to assess the rate and determine measures of SSI burden in Bangladesh

Common Organisms for Surgical Site Infections in Bangladesh

**Figure:**
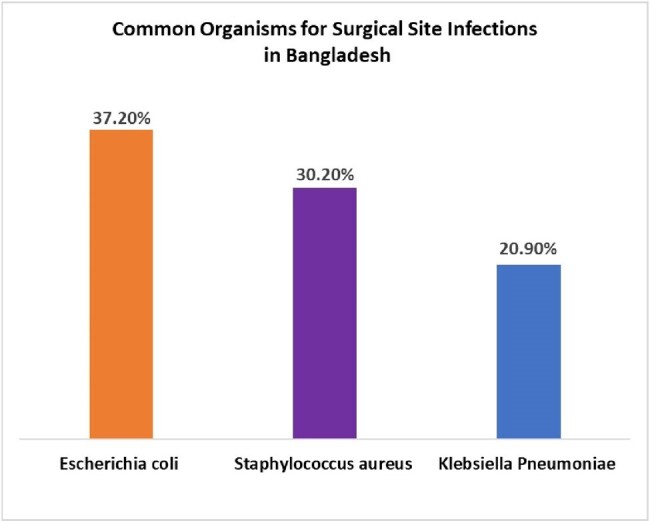

Common Organisms for Surgical Site Infections in Bangladesh

**Methods:**

From January to August 2021, we piloted SSI surveillance in four tertiary care hospitals. Participants included undergoing cesarean section (C-section) or abdominal surgery. We enrolled SSI cases if any infection occurred within 30 days of the surgery and contained the skin, subcutaneous tissue and/or deep soft tissue, in or on any part of the anatomy. We also collected data on symptoms, antibiotic consumption, diagnosis, and treatment

**Results:**

We enrolled 1,760 patients, with a majority (75.7%) undergoing C-sections. Within 30 days of surgery, 33.2% (585) of patients developed SSI symptoms. Abdominal surgery patients had a higher rate of SSI (40.9%, 175/426) compared to C-section patients (30.7%, 410/1334). Most patients (90.9%, 532/585) acquired SSI symptoms during hospital stay. Two-thirds (66.4%) of patients were prescribed antibiotics on admission, and 70.1% were given surgical antibiotic prophylaxis. Rates of SSI did not differ between those who received antibiotics or not. The most frequently prescribed antibiotics were ceftriaxone 57.5%, on admission and the combination of ceftriaxone and metronidazole (84.1%) in postoperative patients. Only 10. 7% (63/585) of participants performed wound cultures, of which 76.2% (48/63) demonstrated growth. The most frequently reported organisms were Escherichia coli (37.2%), Staphylococcus aureus (30.2%), and Klebsiella pneumoniae (20.9%).

**Conclusion:**

Further study of the factors contributing to this high incidence and timing for surgical antibiotic prophylaxis administration is needed. The high rate of antibiotic consumption also suggests a need for examination of whether appropriate treatment is being administered and/or if antibiotic prophylaxis is targeting the correct organisms. Additionally, strengthening IPC practices, establishing antimicrobial stewardship programs, and updating antibiotic guidelines could help prevent SSIs and AMR in Bangladeshi hospitals

**Disclosures:**

**All Authors**: No reported disclosures

